# Antibiotic prophylaxis with piperacillin–tazobactam reduces organ/space surgical site infection after pancreaticoduodenectomy: a retrospective and propensity score-matched analysis

**DOI:** 10.1186/s12885-024-11955-x

**Published:** 2024-02-23

**Authors:** Yifei Yang, Jianjie Sheng, Zhenghua Cai, Linxi Zhu, Chenglin Lu, Liang Mao, Yudong Qiu, Xu Fu

**Affiliations:** 1 Division of Pancreatic Surgery, Department of General Surgery, Nanjing Drum Tower Hospital, the Affiliated Hospital of Medical School, Nanjing University, Nanjing, 210008 China; 2 Division of Pancreatic Surgery, Department of General Surgery, Nanjing Drum Tower Hospital Clinical College of Nanjing University of Chinese Medicine, Nanjing, 210008 China; 3 Department of General Surgery, Pancreatic Disease Center, Nanjing Drum Tower Hospital, The Affiliated Hospital of Nanjing University Medical School, Nanjing, 210002 China

**Keywords:** Pancreaticoduodenectomy, Prophylactic antibiotic, Surgical site infection, Piperacillin–tazobactam

## Abstract

**Background:**

The occurrence of surgical site infection (SSI) after pancreaticoduodenectomy (PD) is still relatively high. The aim of this retrospective study is to evaluate the efficacy of piperacillin-tazobactam as perioperative prophylactic antibiotic on organ/space SSI for patients underwent PD.

**Methods:**

Four hundred seven consecutive patients who underwent PD between January 2018 and December 2022 were enrolled and analyzed retrospectively. The univariate and multivariate analysis were used to identify independent risk factors of organ/space SSI. Postoperative complications were compared between the two groups according to the use of prophylactic antibiotics by a ratio of 1:1 propensity score-matched (PSM) analysis.

**Results:**

Based on perioperative prophylactic antibiotic use, all 407 patients were divided into the ceftriaxone group (*n* = 192, 47.2%) and piperacillin–tazobactam group (*n* = 215, 52.8%). The rate of organ/space SSI was 31.2% with the choice of perioperative antibiotics (OR = 2.837, 95%CI = 1.802–4.465, *P* < 0.01) as one of independent risk factors. After PSM, there were similar baseline characteristics among the groups. Meanwhile, the piperacillin–tazobactam group had a significant lower rate of organ/space SSI compared to the ceftriaxone group both before and after PSM(*P* < 0.05).

**Conclusions:**

The adoption of piperacillin–tazobactam as perioperative prophylaxis for patients underwent PD reduced organ/space SSI significantly.

**Supplementary Information:**

The online version contains supplementary material available at 10.1186/s12885-024-11955-x.

## Introduction

Pancreaticoduodenectomy (PD) is a conventional surgical procedure performed for both benign or malignant disease localized in the pancreatic head or periampullary region with relative high risk of morbidity and mortality [1]. Organ/space surgical site infection (SSI) after PD, which is one of the most frequent postoperative complications as we reported previously [2], sometimes triggers life-threatening complications, such as postoperative fistula (POPF), post-pancreatectomy hemorrhage (PPH), and sepsis [3, 4]. Current guidelines of hepatopancreatobiliary surgery recommend second-generation cephalosporins (cefoxitin or cefotetan) or third-generation cephalosporins (ceftriaxone) as perioperative prophylaxis [5]. As antibiotic resistance increases, the perioperative prophylactic antibiotics provide limited coverage of resistant pathogens, particularly in PD [5, 6]. Yet, the effect of taking broader-spectrum agents as perioperative prophylactic antibiotic regimen for preventing SSI after PD has been investigated in several studies with opposite results [7–10].

Given the high rate of postoperative organ/space SSI and the controversy over perioperative antimicrobial prophylaxis regimens for PD, in current study we evaluate the efficacy of piperacillin-tazobactam as perioperative prophylactic antibiotic for patients underwent PD.

## Methods

### Patients

The medical data of consecutive patients who underwent PD between January 2018 and December 2022 in the Drum Tower Hospital of Nanjing University Medical School were collected retrospectively. The inclusion criteria were as follows: (a)underwent conventional PD or pylorus-preserving PD (PPPD); (b) no evidence of local unresectable or other active cancers at diagnosis, and (c) > 18 years of age. The exclusion criteria were as follows: (a)underwent simultaneous hepatic or colon resection; (b)clinical data were incomplete, and (c)history of neoadjuvant chemotherapy. The study was approved by the Health Research Ethics Board of Drum Tower Hospital of Nanjing University Medical School (2021–271-01).

### Surgical procedures and perioperative management

All PDs were performed by two experienced teams. Standard perioperative managements instead of the choice of prophylactic antibiotics were applied for all patients. The PD procedure and indications of preoperative biliary drainage (PBD) were as previously described [11].

All patients delivered intravenous antibiotics within a 30 min window prior to skin incision [5]. The choice of antibiotic was at the discretion of the operating surgeon. For patients without signs of organ/space SSI [12] during early postoperative period, regardless of whether the ascites culture was positive or negative, intravenous ceftriaxone was administered routinely for 3 days (the operation day and postoperative day 1 and 2) in the ceftriaxone group and piperacillin–tazobactam (4.5 g/500 mg) was given and continued at 13.5 g/1500 mg daily after surgery until postoperative day (POD) 5 intravenous in the piperacillin–tazobactam group. For patients with signs of organ/space SSI, the antibiotics were altered based on bacteriologic profiles and antibiogram of the pathogens in ascites.

Drain fluid test consist of amylase concentration, bacterial smear, and culture were conducted on POD 1,3, 5 and every 2 to 3 days thereafter until drains were removed, regardless of whether the patients underwent CR-POPF and/or SSI. The drainage tubes were removed on POD 7 after the abdominal enhanced computed tomography (CT) conducted on POD 7 showed no evidences of CR-POPF or fluid collection were found.

### Clinical data collection and definition of complication

Demographic data (age, gender, high blood pressure, diabetic mellitus, BMI, preoperative jaundice, preoperative biliary drainage), preoperative laboratory data (alanine aminotransferase, total bilirubin, direct bilirubin, albumin), intraoperative variables (diameter of main pancreatic duct, vessel resection, operating time, volume of blood loss and transfusion), pathological diagnosis were all collected. Postoperative complications were classified according to the Clavien–Dindo classification, with major complications being defined as grade ≥ III [13]. The assessment of SSI which includes incisional and organ/space SSI was based on the Centers for Disease Control and Prevention (CDC) guidelines [12]. Clinically relevant postoperative pancreatic fistula (CR-POPF), and post-pancreatectomy hemorrhage (PPH) were diagnosed according to the International Study Group of Pancreatic Surgery (ISGPS) [14, 15]. Bacteremia, pneumonia and urinary tract infection were all included and diagnosed as previous described [16, 17].

### Statistical analysis

Clinical data was analyzed using SPSS 26.0 software for Windows (SPSS Inc.) was used for clinical data analyses. *χ*^*2*^ test or Fisher’s exact test was performed for categorical variables, which expressed as absolute number and percentage. Normally distributed continuous variables were analyzed by independent *t-*test, which expresses by mean and standard deviation (SD). Mann–Whitney *U* test was applied and showed as median (interquartile range, IQR) for non-normally distributed clinical data. Univariate and multivariate logistic regression analysis of organ/space SSI were completed with the entire cohort of 407 patients. All variables with *P* < 0.1 in univariate analysis entered the multivariate logistic regression model to find out the independent risk factors for organ/space SSI. Odds ratio (OR) and 95% confidence intervals (95%CI) were obtained. For all analyses, *P* < 0.05 was considered as statistics significantly.

To compare the difference of ceftriaxone group and piperacillin–tazobactam group, a 1:1 nearest-neighbor propensity score-matching (PSM) analysis was performed which modifying total bilirubin, direct bilirubin, pancreas consistency, vessel resection, diameter of MPD, operating time, blood loss volume and blood transfusion volume. Caliper matching on propensity score was estimated, and pairs were matched to within a range of 0.2 standard deviation of the logistic model of the propensity score.

## Results

### Patient characteristics

The study enrolled 407 patients during the 5-year study period. They were classified into two groups as piperacillin–tazobactam group (*n* = 215, 52.8%) and ceftriaxone group (*n* = 192, 47.2%) according to the use of perioperative antibiotics. The clinical and baseline characteristics were shown in Table 1. The study included 263(64.6%) men and 144(35.4%) women with the mean age of the entire cohort was 61.7 ± 11.5 years. A total of 146(35.9%) were diagnosed with preoperative jaundice and 100(24.6%) received PBD. 152(37.3%) patients occurred infectious complications which consisted with SSI, bacteremia pneumonia and urinary tract infection. Organ/space SSI was the most common complication after surgery, accounting for 31.2%. 101(24.8%) patients developed CR-POPF and 69(16.9%) patients underwent postoperative major complication (Calvien-Dindo grade ≥ III).

**Table 1:** Clinical characteristic of all patients

**Characteristic**	Total (*n* = 407)
**Age (mean ± SD), years**	61.7 ± 11.5
**Gender, n (%)**	
**Male**	263(64.6%)
**Female**	144 (35.4%)
**BMI (mean ± SD), kg/m**^**2**^	23.3 ± 3.3
**DM, n (%)**	70(17.2%)
**HBP, n (%)**	157(38.6%)
**Jaundice, n (%)**	146(35.9%)
**PBD, n (%)**	100(24.6%)
**ALT (median, IQR), U/L**	44.0(15.6–110.2)
**TB (median, IQR), μmol/L**	15.5(9.2–63.8)
**DB (median, IQR), μmol/L**	4.9(2.2–45.5)
**Alb (mean ± SD), g/L**	39.1 ± 3.0
**Perioperative antibiotics, n (%)**	
**Piperacillin-tazobactam**	215(52.8%)
**Ceftriaxone**	192(47.2%)
**Pathology diagnosis, n (%)**	
**PDAC**	117(28.7%)
**No-PDAC**	290(71.3%)
**Pancreas consistency, n (%)**	
**Hard**	70(17.2%)
**Soft**	337(82.8%)
**Vessel resection, n (%)**	
**Yes**	29(7.1%)
**No**	378(92.9%)
**Diameter of MPD (median, IQR), mm**	3.0(2.0–5.0)
**Operating time (median, IQR), min**	330.0(260.0–420.0)
**Blood loss volume (median, IQR), ml**	400.0(300.0–600.0)
**Blood transfusion (median, IQR), ml**	0.0(0.0–600.0)
**Postoperative complications, n (%)**	
**CR-POPF**	101(24.8%)
**Major complication**	69(16.9%)
**BL**	29(7.1%)
**PPH**	30(7.3%)
**Infectious complications**	152(37.3%)
**Organ/space SSI**	127(31.2%)
**Incision SSI**	20(4.9%)
**Bacteremia**	18(4.4%)
**Pneumonia**	7(1.7%)
**Urinary tract infection**	3(0.7%)
**Duration of hospital stay (median, IQR), days**	26.0(20.0–35.0)
**Postoperative hospital stays (median, IQR), day**	17.0(14.0–25.0)

*SD* standard deviation, *IQR* interquartile, *BMI* body mass index, *DM* diabetes mellitus, *HBP* high blood pressure, *PBD* preoperative biliary drainage, *ALT* alanine aminotransferase, *TB* total bilirubin, *DB* direct bilirubin, *Alb* albumin, *PDAC* pancreatic duct adenocarcinoma, *MPD* main pancreatic duct, *CR-POPF* Clinically relevant postoperative pancreatic fistula, *BL* biliary leakage, *PPH* post-pancreatectomy hemorrhage, *SSI* surgical site infection

### Comparison of postoperative complications

Regardless of whether PBD was performed or not, the incidence of infectious complications (*P* = 0.017 and *P* < 0.001), organ/space SSI (*P* = 0.018 and *P* < 0.001) and organ/space SSI combined with CR-POPF (*P* = 0.023 and *P* = 0.016) were significantly lower in the piperacillin-tazobactam group. As to the severity of complications, the occurrence of CR-POPF (*P* < 0.001), PPH (*P* < 0.001), infectious complications (*P* < 0.001), organ/space SSI (*P* < 0.001), isolated CR-POPF (*P* = 0.016) and organ/space SSI combined with CR-POPF (*P* < 0.001) was significantly higher in patients with major complication (Clavien-Dindo grade ≥ III) (Tables 2 and 3).

**Table 2:** Comparison of postoperative complications according to the preoperative biliary drainage and perioperative antibiotics regimes

	**PBD (*n* = 100)**			**Non-PBD (*n* = 307)**		
**Postoperative complications**	**Ceftriaxone group (*n* = 48)**	**Piperacillin-tazobactam group (*n* = 52)**	***P***	**Ceftriaxone group (*n* = 192)**	**Piperacillin-tazobactam group (*n* = 215)**	***P***
**CR-POPF, n (%)**	15(31.3)	11(21.2)	0.250	36(18.8)	39(18.1)	0.894
**Major complication, n (%)**	11(22.9)	4(7.7)	0.064	31(16.1)	23(10.7)	0.100
**BL, n (%)**	3(6.3)	3(5.8)	1.000	3(1.6)	13(6.0)	0.829
**PPH, n (%)**	3(6.3)	5(9.6)	0.802	8(4.2)	14(6.5)	0.37
**Infectious complications, n (%)**	28(58.3)	18(34.7)	0.017	65(33.9)	41(19.1)	< 0.001
**Organ/space SSI, n (%)**	26(54.2)	16(30.7)	0.018	55(28.6)	30(13.9)	< 0.001
**Incision SSI, n (%)**	2(4.2)	2(3.8)	1.000	7(3.6)	9(4.2)	1.000
**Bacteremia, n (%)**	3(6.3)	2(3.8)	0.927	8(4.2)	5(2.3)	0.213
**Pneumonia, n (%)**	0(0.0)	1(1.9)	1.000	4(2.1)	2(0.9)	0.424
**Urinary tract infection**	0(0.0)	0(0.0)	N/A	3(1.6)	0(0.0)	0.102
**Isolated organ/space SSI, n (%)**	13(27.1)	11(21.2)	0.488	27(14.1)	14(6.5)	0.009
**Isolated CR-POPF, n (%)**	2(4.2)	6(11.5)	0.323	8(4.2)	23(10.7)	0.013
**Organ/space SSI with CR-POPF, n (%)**	13(27.1)	5(9.6)	0.023	28(14.6)	16(7.4)	0.016

*PBD* preoperative biliary drainage, *CR-POPF* Clinically relevant postoperative pancreatic fistula, *BL* biliary leakage, *PPH* post-pancreatectomy hemorrhage, *SSI* surgical site infection, *N/A* not available

**Table 3:** Comparison of postoperative complications according to the severity of postoperative complications

Postoperative complications	Clavien–Dindo grade ≥ III (*n* = 69)	Clavien–Dindo grade < III (*n* = 338)	*P*
**CR-POPF, n (%)**	44(63.8)	57(16.9)	< 0.001
**BL, n (%)**	5(7.2)	24(7.1)	0.966
**PPH, n (%)**	21(30.4)	9(2.7)	< 0.001
**Infectious complications, n (%)**	50(72.4)	102(30.2)	< 0.001
**Organ/space SSI, n (%)**	45(65.2)	82(24.3)	< 0.001
**Incision SSI, n (%)**	8(11.6)	12(3.6)	0.005
**Bacteremia, n (%)**	4(5.8)	14(4.1)	0.773
**Pneumonia, n (%)**	2(2.9)	5(1.5)	0.750
**Urinary tract infection**	1(1.4)	2(0.6)	1.000
**Isolated organ/space SSI, n (%)**	13(18.8)	52(15.4)	0.475
**Isolated CR-POPF, n (%)**	32(46.4)	30(8.9)	0.016
**Organ/space SSI with CR-POPF, n (%)**	12(17.4)	27(7.9)	< 0.001

*PBD* preoperative biliary drainage, *CR-POPF* Clinically relevant postoperative pancreatic fistula, *BL* biliary leakage, *PPH* post-pancreatectomy hemorrhage, *SSI* surgical site infection

### Microbiological analysis

The profile of bacteria in intraoperative bile and postoperative drain fluid was listed in Table 4. *K. pneumoniae* is the most common microorganism in intraoperative bile culture (*n* = 20, 15.3%). Other common bacteria were *E. coli* (*n* = 11, 8.4%) and *E. faecalis* (*n* = 8, 6.1%). The most common bacterial species isolated from postoperative drain fluid were *K. pneumoniae* (*n* = 56, 13.8%), followed by *E. faecalis* (*n* = 49, 12.0%), *E. coli* (*n* = 28, 6.9%), *E. faecium* (*n* = 28, 6.9%), *fungus* (*n* = 28, 6.9%), and *E. cloacae* (*n* = 23, 5.7%). Based on the relative high prevalence of the pathogens detected in intraoperative bile and postoperative ascites drainage, the resistance profile of the selected antibiotics/antimycotics was shown in Supplemental Tables S1 and S2. Bacterial resistance was generally elevated in postoperative abdominal drainage fluid compared with intraoperative bile bacterial resistance. Targeting bacteria in postoperative peritoneal drainage, subgrouped according to the perioperative antibiotic regimen, bacterial resistance in the piperacillin-tazobactam group was essentially similar to that in the ceftriaxone group.

**Table 4:** Microorganisms cultured from intraoperative bile and drainage fluid after pancreaticoduodenectomy

Microorganisms	Intraoperative bile culture (*n* = 131)	Postoperative drain fluid culture (*n* = 407)
*K. pneumoniae*, n (%)	20(15.3)	56(13.8)
*E. faecalis*, n (%)	8(6.1)	49(12.0)
*E. coli*, n (%)	11(8.4)	28(6.9)
*E. faecium*, n (%)	0(0.0)	28(6.9)
*Fungus*, n (%)	3(2.3)	28(6.9)
*E. cloacae*, n (%)	7(5.3)	23(5.7)
*A. baumannii*, n (%)	5(3.8)	20(4.9)
*S. aureus*, n (%)	1(0.8)	14(3.4)
*P. aeruginosa*, n (%)	2(1.5)	13(3.2)

### Risk factors for organ/space SSI

In univariate analysis, gender (OR = 1.676, 95%CI = 1.060–2.647, *P* = 0.027), PBD (OR = 1.891, 95%CI = 1.183–3.024, *P* = 0.008), the choice of perioperative antibiotic (ceftriaxone vs. piperacillin–tazobactam) (OR = 2.681, 95%CI = 1.737–4.137 *P* < 0.001) and the diameter of main pancreatic duct (MPD) (OR = 0.872 95%CI = 0.777–0.979, *P* = 0.021) were associated with the development of organ/space SSI significantly. In multivariate analysis, gender (OR = 1.956, 95%CI = 1.204–3.177 *P* = 0.007), PBD (OR = 1.730, 95%CI = 1.041–2.875, *P* = 0.034), the choice of perioperative antibiotic (ceftriaxone vs. piperacillin–tazobactam) (OR = 2.837, 95%CI = 1.802–4.465, *P* < 0.001) and the diameter of MPD (OR = 0.879, 95%CI = 0.776–0.995 *P* = 0.041) were also the independent risk factors of organ/space SSI (Table 5).

**Table 5:** Risk factors of organ/space SSI: Univariate and multivariate logistic regression analysis

Variables	Univariate analysis		Multivariate analysis	
	**OR (95%CI)**	***P***	**OR (95%CI)**	***P***
**Age**	0.988(0.970–1.006)	0.198		
**Gender**	1.676(1.060–2.647)	0.027	1.956(1.204–3.177)	0.007
**BMI**	0.971(0.914–1.033)	0.354		
**DM**	1.163(0.660–2.050)	0.602		
**HBP**	1.333(0.870–2.043)	0.187		
**Jaundice**	1.307(0.848–2.014)	0.225		
**PBD**	1.891(1.183–3.024)	0.008	1.730(1.041–2.875)	0.034
**ALT**	0.999(0.998–1.001)	0.481		
**TB**	1.002(0.999–1.004)	0.237		
**DB**	1.003(1.000–1.007)	0.087	1.003(0.999–1.008)	0.107
**Alb**	0.935(0.872–1.002)	0.056		
**Perioperative antibiotics**	2.681(1.737–4.137)	< 0.001	2.837(1.802–4.465)	< 0.001
**Pathology diagnosis**	0.773(0.481–1.242)	0.287		
**Pancreas consistency**	0.987(0.567–1.720)	0.964		
**Vessel resection**	1.614(0.747–3.489)	0.223		
**Diameter of MPD**	0.872(0.777–0.979)	0.021	0.879(0.776–0.995)	0.041
**Operating time**	1.001(0.999–1.003)	0.185		
**Blood loss volume**	1.000(0.999–1.000)	0.467		
**Blood transfusion**	1.000(1.000–1.001)	0.173		

*SSI* surgical site infection, *BMI* body mass index, *DM* diabetes mellitus, *HBP* high blood pressure, *PBD* preoperative biliary drainage, *ALT* alanine aminotransferase, *TB* total bilirubin, *DB* direct bilirubin, *Alb* albumin, *MPD* main pancreatic duct, *CI* confidence interval, *OR* odds ratio

### Propensity score-matched analysis

As shown in Table 6, patients treated with piperacillin–tazobactam had lower level of total bilirubin (TB) and direct bilirubin (DB). At the same time, patients in the ceftriaxone group had higher level of operating time, blood loss volume and blood transfusion volume compared with the piperacillin–tazobactam group. Furthermore, the rate of vessel resection and consistency of pancreas showed statistical difference.

**Table 6:** Baseline characteristics in the unmatched and matched group according to the perioperative antibiotics

Variables	Before PS matching			After PS matching		
	**Ceftriaxone group (*n* = 192)**	**Piperacillin-tazobactam group (*n* = 215)**	***P***	**Ceftriaxone group (*n* = 110)**	**Piperacillin-tazobactam group (*n* = 110)**	***P***
**Age (mean ± SD), years**	61.1 ± 11.7	62.4 ± 11.4	0.240	61.7 ± 11.8	61.1 ± 11.7	0.717
**Gender, n (%)**			0.398			
**Male**	120(62.5)	143(66.5)		60	68	0.274
**Female**	72(37.5)	72(33.5)		50	42	
**BMI (mean ± SD), kg/m**^**2**^	23.4 ± 3.3	23.4 ± 3.4	0.934	23.3 ± 3.2	23.8 ± 3.7	0.28
**DM, n (%)**	39(20.3)	31(14.4)	0.116	19(17.3)	12(10.9)	0.175
**HBP, n (%)**	71(36.9)	86(40.0)	0.532	41(37.3)	38(34.5)	0.673
**Jaundice, n (%)**	64(33.3)	82(38.1)	0.313	39(35.5)	30(27.3)	0.191
**PBD, n (%)**	48(25.0)	52(24.2)	0.849	27(24.5)	22(20.0)	0.418
**ALT (median, IQR), U/L**	41.5(15.6–110.2)	47.1(15.9–109.3)	0.799	58.1(16.5–120.0)	35.4(15.4–79.9)	0.072
**TB (median, IQR), μmol/L**	13.9(8.8–46.5)	18.5(9.6–90.9)	0.034	15.0(9.4–52.9)	13.9(8.9–56.4)	0.572
**DB (median, IQR), μmol/L**	4.5(2.0–33.4)	6.2(2.3–64.9)	0.062	5.5(2.3–39.9)	4.0(2.1–39.8)	0.469
**Alb (mean ± SD), g/L**	39.3 ± 2.9	38.9 ± 3.1	0.170	38.9 ± 2.9	39.3 ± 3.1	0.277
**Pathology diagnosis, n (%)**			0.357			0.057
**PDAC**	51(26.6)	66(30.7)		20(18.2)	32(29.1)	
**No-PDAC**	141(73.4)	149(69.3)		90(81.8)	78(70.1)	
**Pancreas consistency, n (%)**			0.009			0.186
**Hard**	43(22.4%)	27(12.6%)		13(11.8)	20(18.2)	
**Soft**	149(77.6%)	188(87.4%)		97(88.2)	90(81.8)	
**Vessel resection, n (%)**			0.005			0.332
**Yes**	21(10.9)	8(3.7)		3(2.7)	7(6.6)	
**No**	171(89.1)	207(96.3)		107(97.3)	103(93.4)	
**Diameter of MPD (median, IQR), mm**	2.5(2.0–5.0)	3.0(2.0–5.0)	0.008	3.0(2.0–5.0)	3.0(2.0–4.3)	0.723
**Operating time (median, IQR), min**	390.0(326.3–445.0)	280.0(240.0–350.0)	< 0.001	340.0(295.0–420.0)	330(270.0–410.0)	0.313
**Blood loss volume (median, IQR), ml**	425.0(300.0–637.5)	300.0(300.0–500.0)	0.001	400.0(300.0–600.0)	400.0(287.5–600.0)	0.472
**Blood transfusion (median, IQR), ml**	0.0(0.0–775.0)	0.0(0.0–600.0)	0.040	0.0(0.0–737.5)	0.0(0.0–600.0)	0.572

*SD* standard deviation, *IQR* interquartile, *BMI* body mass index, *DM* diabetes mellitus, *HBP* high blood pressure, *PBD* preoperative biliary drainage, *ALT* alanine aminotransferase, *TB* total bilirubin, *DB* direct bilirubin, *Alb* albumin, *PDAC* pancreatic duct adenocarcinoma, *MPD* main pancreatic duct

In order to adjust the differences of baseline variables in each group, a 1:1 nearest-neighbor propensity score matching (PSM) analysis was conducted. After PSM, a balanced cohort included the piperacillin–tazobactam group as observational group (110 patients) and the ceftriaxone group as the control group (110 patients). All baseline characteristics were comparable after PSM.

### Postoperative complications according to PSM

After PSM, organ/space SSI occurred in 43(39.1%) patients in the ceftriaxone group and 21(19.1%) patients in the piperacillin–tazobactam group (*P* = 0.001; Table 7). Both before and after PSM, the major complication occurred more frequently in the ceftriaxone group significantly. Furthermore, the rates of CR-POPF, BL, PPH, incisional SSI, bacteremia, pneumonia, and urinary tract infection were comparable between the two groups (Table 7).

**Table 7:** Postoperative mortality and morbidity according to the perioperative antibiotics

Variables	Before PS matching			After PS matching		
	**Ceftriaxone group (*n* = 192)**	**Piperacillin-tazobactam group (*n* = 215)**	***P***	**Ceftriaxone group (*n* = 110)**	**Piperacillin-tazobactam group (*n* = 110)**	***P***
**CR-POPF**	51(26.5)	50(23.2)	0.441	28(25.5)	26(23.6)	0.754
**Major complication**	42(21.5)	27(12.7)	0.012	28(25.5)	16(14.5)	0.043
**BL**	13(6.7)	16(5.4)	0.793	8(7.3)	9(8.2)	0.801
**PPH**	11(5.7)	19(8.8)	0.231	6(5.5)	9(8.2)	0.422
**Infectious complications**	93(48.4)	59(27.4)	< 0.001	51(46.4)	29(26.4)	0.002
**Organ/space SSI**	81(42.1)	46(21.3)	< 0.001	43(39.1)	21(19.1)	0.001
**Incision SSI**	9(4.6)	11(5.1)	0.842	6(5.5)	8(7.3)	0.581
**Bacteremia**	11(5.7)	7(3.2)	0.226	7(6.4)	3(2.7)	0.332
**Pneumonia**	4(2.1)	3(1.3)	0.880	2(1.8)	0(0.0)	0.498
**Urinary tract infection**	3(1.6)	0(0.0)	0.104	3(2.7)	0(0.0)	0.247

*CR-POPF* Clinically relevant postoperative pancreatic fistula, *BL* biliary leakage, *PPH* post-pancreatectomy hemorrhage, *SSI* surgical site infection, *PS* propensity score

## Discussion

In this study, the occurrence of postoperative major complication (Clavien–Dindo grade ≥ III), infectious complication, and organ/space SSI were 16.9%, 37.3%, and 31.2%, respectively, and consistent with previous studies [10, 17, 18]. We also identified that the choice of perioperative prophylactic antibiotic was one of the independent risk factors for organ/space SSI. Meanwhile, *K. pneumonia, E. coli* and *E. faecalis* were the most frequently isolated pathogens in both intraoperative bile and postoperative drain fluids. In addition, we conducted an additional analysis by propensity score-matching (PSM) to lessen the bias of baseline variables between two groups. Both before and after PSM, the occurrence of major complication, infectious complications and organ/space SSI were significantly higher in the ceftriaxone group. Meanwhile, the additional therapeutic antibiotics administration rates after PD according to clinical symptoms of organ/space SSI in piperacillin-tazobactam and ceftriaxone group were 25.5% and 59.9%, respectively.

Despite the dramatic improvements in surgical techniques of PD, postoperative morbidity has persistently remained high. Given that organ/space SSI is the most frequent cause of postoperative complications, which may trigger subsequent events such as CR-POPF, sepsis, readmission or even death, this retrospective study’s findings are notable. As we reported previously and showed in this study, PBD was substantially correlated with postoperative infectious complications especially organ/space SSI [2, 11, 19]. This invasive operation destroys the function of Oddi’s sphincter, increase the contamination of surgical field with bile which may contain microbes resistant to ceftriaxone after the resection of common bile duct [20, 21].

The microbiology of ascites after PD, which carries a significant risk of digestive anastomotic leakage and abdominal infection, is still challenging. Several reports had indicated that *Enterococcus*, *Enterobacter* and *Klebsiella* species were the predominant organisms isolated from SSI after PD [21–23]. Our previous research identified *K. pneumoniae, E. faecalis and S. haemolyticus* were the most frequently isolated bacteria in bile culture [24]. Moreover, the most common bacterial species isolated from the drainage fluid in current study were *K. pneumoniae,* followed by *E. faecalis, E. coli and E. faecium,* which were almost consistent with the existing studies leading us to speculate that intraoperative bile contamination might correlate and promote the organ/space SSI [18, 25]. This speculation is supported indirectly by the fact that a number of studies had demonstrated that specific antibiotic based on bile culture was effective on reducing the incidence of organ/space SSI [26–28]. We identified *K. pneumoniae* had negative impacts on organ/space SSI, major complications, CR-POPF based on past research [11]. The study focused on the anastomoses of the digestive tract had implicated collagenase-producing pathogens, such as *Enterococcus*, in the formation of anastomotic leakage which may result in subsequent organ/space SSI [29–31]. The administration of piperacillin-tazobactam may cover more pathogens especially *Enterococcus* which contaminate the surgical field during the operation than ceftriaxone, thus reducing the incidence of organ/space SSI. The reduced rates of major complication and infectious complications are likely to be related to the percutaneous drainage placement, reoperation and postoperative mortality.

The regimes recommended by guidelines for perioperative prophylactic antibiotic are variable. Additionally, there is a lack of compliance with guidelines. For the purpose of decreasing the occurrence of organ/space SSI, piperacillin-tazobactam was utilized as prophylactic antibiotic. Patients underwent pancreatic resection especially pancreaticoduodenectomy, however, still lack a clear indication of perioperative prophylactic antibiotic. The Japanese investigation conducted by Kimura et al. revealed significant variation in the pancreaticoduodenectomy perioperative prophylactic scheme, including the application of ampicillin, various cephalosporin classes, cefoperazone-sulbactam, and carbapenems [32]. At the same time, the study identified substantial variation in the time requisite for surgical prophylaxis, in the range of 1 to 14 postoperative days. As the increasing antibiotic resistance of the organisms colonizing on the bile duct, institutional data and policy were revised according to earlier researches. According to a recent meta-analysis conducted by Droogh et al. that included 8 studies, prolonged prophylactic antibiotic usage for patients who underwent PBD before surgery significantly reduced the incidence of abdominal infections [33]. In addition, the article suggests that the antibiotic resistance of patients undergoing perioperative or long-term prevention was comparable. Pastena et al. reported adopting antibiotic prophylaxis on the basis of piperacillin-tazobactam was associated with reducing postoperative SSI [10]. The randomized controlled study conducted by D’Angelica et al. found that the use of piperacillin-tazobactam as perioperative prophylactic antibiotic for PD was effective in reducing several postoperative complications including organ/space SSI [1]. The data gathered as a result of current study supported the hypothesis that organ/space SSI can be reduced by the application of the proper antibiotic prophylaxis, such as piperacillin-tazobactam.

To the best of our knowledge, the present study is the first retrospective cohort utilizing PSM for identifying the impact of piperacillin-tazobactam about the postoperative complications especially organ/space SSI. After PSM, the introduction of piperacillin-tazobactam decreases the development several postoperative complications, especially organ/space SSI. Furthermore, according to the results of current study, postoperative drainage bacterial resistance showed an elevated trend compared to intraoperative bile. Meanwhile, abdominal isolates resistance in patients who introduced piperacillin-tazobactam as perioperative prophylactic antibiotic was basically similar compared to ceftriaxone, which consistent with Droogh et al. [33]. The retrospective cohort study conducted by Tarvainen et al. revealed that the resistance of second-generation cephalosporin in intraoperative bile was common in patients who underwent PBD. They pointed out that the take broad-spectrum antibiotics as perioperative prophylaxis may be beneficial for these high-risk patients [34]. These indicate that the emergence of multi-drug resistant microorganisms, broader spectrum antibiotics such as piperacillin-tazobactam can be adapted as prophylactic antibiotic especially after PD, especially for those who underwent PBD.

The present study has several limitations. First, it was a single center retrospective study accompanied by unavoidable biases, such as year of surgery and the surgeon's choice of antibiotics, even though the patients in the time period of current study were under a relatively fixed perioperative treatment regimen at our center. Further multicenter and randomize controlled trails are indispensable to validate the impact of piperacillin-tazobactam on organ/space SSI. Second, even though we utilized propensity score to reduce the effect of confounding variables on the results of the study, there were still some confounders that remained unobserved. At the same time, the introducing of propensity scores raised the issue of potentially dropping cases (i.e., dropping participants who cannot be matched), which lead to imperfect matching (i.e., missing cases and reduced sample size). Therefore, further randomized controlled studies are needed to confirm the results of this study. Third, the present study design cannot determine whether the reduction in organ/space SSI was related to the duration of antibiotic administration. A further prospective trial investigating the duration of antibiotic administration on postoperative complications is currently underway.

In conclusion, the outcomes of our study showed that the regime of perioperative antibiotics was an independent risk factor of organ/space SSI after PD. At the same time, the patients take piperacillin-tazobactam as prophylactic antibiotic experience a lower rate of organ/space SSI even after PSM.

### Supplementary Information

**Additional file 1: Supplemental Table S1.** The profile of antibiotics resistance in microorganisms cultured from intraoperative bile. **Supplemental Table S2.** The profile of antibiotics resistance in microorganisms cultured from postoperative drainage fluid.

## Data Availability

The datasets generated and/or analyzed during the present study are not publicly available due to patient privacy concerns, but are available from the corresponding author upon reasonable request.
