# Supplementary material for: Antibiotic prophylaxis with piperacillin–tazobactam reduces organ/space surgical site infection after pancreaticoduodenectomy: a retrospective and propensity score-matched analysis
Source: BMC Cancer. 2024 Feb 23;24:251. doi: 10.1186/s12885-024-11955-x (PMC10885369; doi:10.1186/s12885-024-11955-x)
Supplement: Supplementary file 1 — Additional file 1: Supplemental Table S1. The profile of antibiotics resistance in microorganisms cultured from intraoperative bile. Supplemental Table S2. The profile of antibiotics resistance in microorganisms cultured from postoperative drainage fluid. [file 12885_2024_11955_MOESM1_ESM.docx]

**Supplemental Table S1 The profile of antibiotics resistance in microorganisms cultured from intraoperative bile**

| **Gram-negative bacteria** | | | | | | | | | | | | |
| --- | --- | --- | --- | --- | --- | --- | --- | --- | --- | --- | --- | --- |
|  | **Ceftazidime** | | **Cefotaxime** | | **Piperacillin-tazobactam** | | **Imipenem** | | **Levofloxacin** | | **Tigecycline** | |
|  | **Ceftriaxone group** | **Piperacillin-tazobactam group** | **Ceftriaxone group** | **Piperacillin-tazobactam group** | **Ceftriaxone group** | **Piperacillin-tazobactam group** | **Ceftriaxone group** | **Piperacillin-tazobactam group** | **Ceftriaxone group** | **Piperacillin-tazobactam group** | **Ceftriaxone group** | **Piperacillin-tazobactam group** |
| ***K. pneumoniae*, n (%)** | 0(0.0) | 3(42.9) | 0(0.0) | 2(28.6) | (0.0) | 2(28.6) | 0(0.0) | 0(0.0) | 1(7.7) | 4(57.1) | 0(0.0) | 0(0.0) |
| ***E. coli*, n (%)** | 1(14.3) | 0(0.0) | 0(0.0) | 1(25.0) | 0(0.0) | 0(0.0) | 0(0.0) | 0(0.0) | 2(28.6) | 2(50.0) | 0(0.0) | 0(0.0) |
| ***E. cloacae*, n (%)** | 3(60.0) | 0(0.0) | 2(40.0) | 0(0.0) | 2(40.0) | 0(0.0) | 0(0.0) | 0(0.0) | 1(20.0) | 0(0.0) | 0(0.0) | 0(0.0) |
| ***A. baumannii*, n (%)^a^** | 0(0.0) | 1(50.0) | - | - | 1(33.3) | 0(0.0) | 0(0.0) | 0(0.0) | 0(0.0) | 0(0.0) | 0(0.0) | 0(0.0) |
| ***P. aeruginosa*, n (%)^b^** | - | - | 0(0.0) | 1(100.0) | 0(0.0) | 1(100.0) | 0(0.0) | 1(100.0) | 0(0.0) | 1(100.0) | - | - |
| **Gram-positive bacteria** | | | | | | | | | | | | |
|  | **Penicillin** | | **Levofloxacin** | | **Vancomycin** | | **Linezolid** | | **Teicoplanin** | | **Tigecycline** | |
|  | **Ceftriaxone group** | **Piperacillin-tazobactam group** | **Ceftriaxone group** | **Piperacillin-tazobactam group** | **Ceftriaxone group** | **Piperacillin-tazobactam group** | **Ceftriaxone group** | **Piperacillin-tazobactam group** | **Ceftriaxone group** | **Piperacillin-tazobactam group** | **Ceftriaxone group** | **Piperacillin-tazobactam group** |
| ***E. faecalis*, n (%)** | 0(0.0) | 0(0.0) | 0(0.0) | 1(33.3) | 0(0.0) | 0(0.0) | 0(0.0) | 0(0.0) | 0(0.0) | 0(0.0) | 0(0.0) | 0(0.0) |
| ***E. faecium*, n (%)^c^** | - |  | - |  | - |  | - |  | - |  | - |  |
| ***S. aureus*, n (%)^d^** | 1(100.0) | - | 0(0.0) | - | 0(0.0) | - | 0(0.0) | - | 0(0.0) | - | 0(0.0) | - |
|  | **Fluconazole** | | | | **Itraconazole** | | | | **Voriconazole** | | | |
|  | **Ceftriaxone group** | | **Piperacillin-tazobactam group** | | **Ceftriaxone group** | | **Piperacillin-tazobactam group** | | **Ceftriaxone group** | | **Piperacillin-tazobactam group** | |
| ***Fungus*, n (%)** | 0(0.0) | | 0(0.0) | | 0(0.0) | | 0(0.0) | | 0(0.0) | | 0(0.0) | |

^a^ No resistance testing of *A. baumannii* to cefotaxime

^b^ No resistance testing of *P. aeruginosa* to ceftazidime and tigecycline

^c^ No *E. faecium* was isolated from intraoperative bile both in the Ceftriaxone and piperacillin tazobactam group.

^d^ No *S. aureus* was isolated from the intraoperative bile of patients in the piperacillin tazobactam group

**Supplemental Table S2 The profile of antibiotics resistance in microorganisms cultured from postoperative drainage fluid**

| **Gram-negative bacteria** | | | | | | | | | | | | |
| --- | --- | --- | --- | --- | --- | --- | --- | --- | --- | --- | --- | --- |
|  | **Ceftazidime** | | **Cefotaxime** | | **Piperacillin-tazobactam** | | **Imipenem** | | **Levofloxacin** | | **Tigecycline** | |
|  | **Ceftriaxone group** | **Piperacillin-tazobactam group** | **Ceftriaxone group** | **Piperacillin-tazobactam group** | **Ceftriaxone group** | **Piperacillin-tazobactam group** | **Ceftriaxone group** | **Piperacillin-tazobactam group** | **Ceftriaxone group** | **Piperacillin-tazobactam group** | **Ceftriaxone group** | **Piperacillin-tazobactam group** |
| ***K. pneumoniae*, n (%)** | 14(53.8) | 17(56.6) | 9(34.6) | 7(23.3) | 9(34.6) | 16(53.3) | 5(19.2) | 4(13.3) | 12(46.2) | 14(46.7) | 5(19.2) | 3(10.0) |
| ***E. coli*, n (%)** | 9(52.9) | 7(63.4) | 5(29.4) | 5(45.5) | 5(29.4) | 4(36.4) | 0(0.0) | 1(9.1) | 14(82.4) | 10(90.9) | 0(0.0) | 0(0.0) |
| ***E. cloacae*, n (%)** | 8(53.3) | 4(50.0) | 6(40.0) | 1(12.5) | 7(46.7) | 5(62.5) | 3(20.0) | 0(0.0) | 7(46.7) | 2(25.0) | 1(6.7) | 0(0.0) |
| ***A. baumannii*, n (%)^a^** | 8(50.0) | 1(25.0) | - | - | 5(31.3) | 1(25.0) | 4(25.0) | 1(25.0) | 4(25.0) | 1(25.0) | 2(12.5) | 1(25.0) |
| ***P. aeruginosa*, n (%)^b^** | - | - | 1(25.0) | 2(22.2) | 0(0.0) | 2(22.2) | 1(25.0) | 3(33.3) | 1(25.0) | 2(22.2) | - | - |
| **Gram-positive bacteria** | | | | | | | | | | | | |
|  | **Penicillin** | | **Levofloxacin** | | **Vancomycin** | | **Linezolid** | | **Teicoplanin** | | **Tigecycline** | |
|  | **Ceftriaxone group** | **Piperacillin-tazobactam group** | **Ceftriaxone group** | **Piperacillin-tazobactam group** | **Ceftriaxone group** | **Piperacillin-tazobactam group** | **Ceftriaxone group** | **Piperacillin-tazobactam group** | **Ceftriaxone group** | **Piperacillin-tazobactam group** | **Ceftriaxone group** | **Piperacillin-tazobactam group** |
| ***E. faecalis*, n (%)** | 3(9.7) | 2(11.1) | 9(29.0) | 4(22.2) | 0(0.0) | 0(0.0) | 2(6.5) | 0(0.0) | 0(0.0) | 0(0.0) | 0(0.0) | 0(0.0) |
| ***E. faecium*, n (%)** | 12(85.7) | 13(92.9) | 12(85.7) | 13(92.9) | 0(0.0) | 0(0.0) | 0(0.0) | 0(0.0) | 0(0.0) | 0(0.0) | 0(0.0) | 0(0.0) |
| ***S. aureus*, n (%)** | 8(88.9) | 5(100.0) | 3(33.3) | 1(20.0) | 0(0.0) | 0(0.0) | 0(0.0) | 0(0.0) | 0(0.0) | 0(0.0) | 0(0.0) | 0(0.0) |
|  | **Fluconazole** | | | | **Itraconazole** | | | | **Voriconazole** | | | |
|  | **Ceftriaxone group** | | **Piperacillin-tazobactam group** | | **Ceftriaxone group** | | **Piperacillin-tazobactam group** | | **Ceftriaxone group** | | **Piperacillin-tazobactam group** | |
| ***Fungus*, n (%)** | 3(20.0) | | 0(0.0) | | 3(20.0) | | 1(7.7) | | 2(13.3) | | 0(0.0) | |

^a^ No resistance testing of *A. baumannii* to cefotaxime

^b^ No resistance testing of *P. aeruginosa* to ceftazidime and tigecycline
